# West Nile virus detected in louse flies (Diptera: Hippoboscidae) collected from rehabilitated raptors in South Carolina

**DOI:** 10.1186/s13071-026-07428-8

**Published:** 2026-05-05

**Authors:** Katherine M. Brown, Martha Weber, Kia Zellars, Lidia Gual-Gonzalez, Madeleine M Meyer-Torelli, Michael J. Skvarla, Wayne Knee, Will K. Reeves, Melissa S. Nolan

**Affiliations:** 1 Department of Epidemiology and Biostatistics, Arnold School of Public Health, University of South Carolina, Columbia, SC 29208 USA; 2 Institute for Infectious Disease Translational Research, University of South Carolina, Columbia, SC 29208 USA; 3 Riverbanks Zoo and Garden, Columbia, SC 29210 USA; 4 Department of Public Health Sciences, College of Behavioral, Social and Health Sciences, Clemson University, Clemson, SC 29634 USA; 5 Department of Entomology, College of Agricultural Sciences, Penn State University, University Park, PA 16802 USA; 6 Arachnids and Nematodes, Canadian National Collection of Insects, Agriculture and Agri-Food Canada, Ottawa, ON K1A 0C6 Canada; 7 C.P. Gillette Museum of Arthropod Diversity Fort Collins, Fort Collins, CO 80523 USA

## Abstract

**Background:**

Louse flies (Diptera: Hippoboscidae), commonly known as keds or flat flies, are a family of insects that are obligate ectoparasites of birds and mammals, with 216 species documented worldwide. Approximately 75% of the species in the Hippoboscidae family infest birds, while the remaining 25% infest various mammals. Despite the diversity within this family, understanding of their prevalence, vector competencies, and geographic distribution is still quite limited.

**Methods:**

From August 2023 to December 2024, louse flies were collected from wild raptors brought for rehabilitation to the Riverbanks Zoo and Garden in Columbia, South Carolina, USA, as part of a surveillance study. Ectoparasites were removed and stored in 75% ethanol (initially) and DNA/RNA Shield (later), and comprehensive health reports were obtained for all admitted birds of prey. Zoo staff collected saliva samples from a subset of raptors with attached flies, and all samples were tested for West Nile virus (WNV) via real-time polymerase chain reaction (RT-PCR).

**Results:**

Over the 16-month study, approximately 75 louse flies were collected from 21 birds representing six avian species. The most louse flies collected were *Icosta americana *(Leach, 1817), while only two samples contained *Icosta rufiventris*, a species previously unreported in South Carolina. Louse flies were pooled for RT-PCR testing, yielding 36 fly pools with an overall 42% WNV positivity rate. Twelve raptor saliva samples were also available for WNV testing; of these, four (33%) tested positive.

**Conclusions:**

This represents the first WNV analysis in avian louse flies collected in South Carolina and, to our knowledge, includes the first report of *Icosta rufiventris* in the region. Discordant results between raptor WNV infection and the presence of attached ectoparasite WNV underscore a major knowledge gap within the scientific community regarding the role of avian louse flies in the WNV transmission cycle. Further research is needed to determine whether louse flies represent a potential WNV vector for birds.

**Graphical Abstract:**

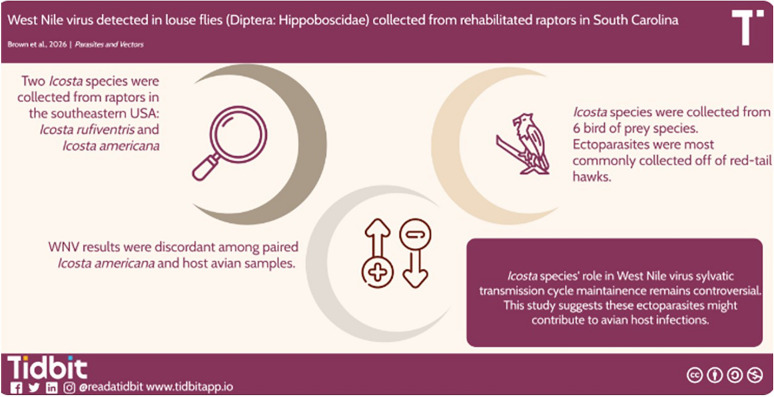

**Supplementary Information:**

The online version contains supplementary material available at 10.1186/s13071-026-07428-8.

## Background

Louse flies (Diptera: Hippoboscidae), commonly referred to as keds or flat flies, are obligate ectoparasites of birds and mammals [1]. With at least 216 species documented globally, Hippoboscidae infest a wide range of domesticated and sylvatic animals; however, most species prefer mammalian or avian hosts [2]. Approximately 75% of species are ectoparasites of birds, while the remaining 25% of species parasitize mammals [3]. Louse fly species infest various avian hosts, such as owls, hawks, vultures, osprey, warblers, pelicans, and others [3]. Some species appear to be quite specific to their bird hosts, with *Icosta americana* only known to infest hawks, owls, grouse, and turkeys [3]. In a recent study of louse flies, highlighting contemporary North American and European records, researchers found that *I. americana* was the most abundant avian louse fly species, representing 91% of their collections [4].

*Icosta americana* (Leach, 1817) was a subject of inquiry following the detection of West Nile virus (WNV) RNA in owls during a Canadian WNV outbreak [5]. Other evidence shows WNV RNA found in *I. americana* collected from New Jersey raptors [6]. Half of the *I. americana* specimens in the study showed no evidence of a bloodmeal, suggesting that the virus could have been present in the hemocoel and tissues, raising concerns about their potential role as a WNV vector [6]. Despite their unknown vector competence and the fact that avian feeding species dominate Hippoboscids, avian louse flies remain understudied, with limited data on prevalence, vector competence, and geographic distribution [4].

The ecological implications of louse flies as vectors of pathogens in human health are equally understudied. Some bird-feeding Hippoboscids can vector several pathogenic organisms, such as *Haemoproteus columbae* and *Trypanosoma avium* [7, 8]. Wild-caught Hippoboscids have tested positive for human pathogens, including *Rickettsia* species, *Borrelia burgdorferi, Anaplasma phagocytophilum*, and WNV [9, 10]. However, their potential vector competence has yet to be confirmed. Raptors that are hosts of *I. americanum* are extremely susceptible to WNV and are known reservoirs, making potential transmission by *I. americanum* ornithologically relevant [11]. Raptors are an important species for monitoring seasonal WNV circulation and potential new outbreaks [11]. To help address this knowledge gap, we investigated louse fly prevalence in rehabilitated raptors and the presence of WNV RNA in bird saliva and louse flies across South Carolina.

## Methods

### Study design, data collection, and sample processing

As a raptor rehabilitation center, the Riverbanks Zoo and Garden in Columbia, South Carolina, USA, regularly receives public-submitted injured raptors. From August 2023 to December 2024, veterinarian staff collected louse flies and host raptors’ saliva samples brought to the Riverbanks Zoo—raptors often arrive with louse fly infestations, which are removed during their veterinary intake procedure. As louse fly removal is part of routine clinical practice, the study was considered exempt from IACUC review, and administrative approval was obtained by the Riverbanks Zoo and Garden. During the first 3 months, louse flies were collected from raptors and grouped by the individual host into vials containing 75% ethanol. After 3 months, a decision was made to test samples for WNV RNA, which changed the storage method; ethanol was replaced with 2 ml DNA/RNA Shield (Zymo Research Corp., Irvine, CA, USA) to preserve any potential viral RNA. Similarly, following initial WNV detection from the 2023 samples, starting in June 2024, additional collection and testing of raptor host saliva were incorporated in the existing protocol. Raptor saliva and buccal material were collected via sterile foam-tipped applicators and individually stored in 2-ml DNA/RNA Shield vials.

Louse flies collected from each bird were grouped into pools of up to three flies based on date of collection, county of origin, bird species, and louse fly species. Only louse flies inhabiting the same bird were pooled together. Information on the raptor species, collection date, county of origin, distribution of louse flies, and description of the bird’s health were all recorded by zoo staff using a standardized form. All louse flies were transported to the University of South Carolina every other month and stored at − 80 °C.

Louse fly taxonomic identification was performed using the taxonomic keys by authors MJS and WKR [12, 13]. In addition, eight louse flies had attached ectoparasitic mites—these mites were sent to the Canadian National Collection of Insects, Arachnids, and Nematodes for taxonomic identification using acarological keys by author WK [14, 15].

### RNA extraction and purification

Pooled louse fly samples and raptor saliva swab solutions were separately processed using the QIAamp Viral RNA Mini Kit, following the manufacturer's guidelines (Qiagen, Hilden, Germany). A 140-ml aliquot of the saliva swab RNA/DNA Shield solution was processed directly following the manufacturer’s guidelines. Initially, fly pools were placed into individual 2-ml microcentrifuge tubes containing a 5-mm stainless steel bead and 400 µl of Qiagen Buffer AVL. Pooled louse fly samples were homogenized for 3 min at 30 Hz using the TissueLyser II (Qiagen, Hilden, Germany), then rotated and homogenized for an additional 3 min. Homogenized samples were then centrifuged for 4 min at 13,300 RCF to separate nucleic acids from residual solids; 140 µl supernatant was then purified per the QIAamp Viral RNA Mini Kit handbook. Purified RNA and the remaining homogenate were stored at − 80 °C for later use.

### WNV RT-PCR testing

Purified RNA was tested for West Nile virus via real-time polymerase chain reaction (RT-PCR). A 70-bp region of the NY99 WNV whole genome was targeted using the primer/probes described by Lanciotti et al. [16]. Reaction mixtures of 20 µl per reaction were prepared using 5 µl Qiagen 4 × One-Step Viral RT-PCR Master Mix, 0.2 µl reverse transcriptase (Qiagen, Hilden, Germany), 0.8 µl 10 µM WNV_1160, 0.8 µl 10 µM WNV_1129c, 0.4 µl 10 µM FAM probe, 7.8 µl nuclease-free water, and 5 µl template RNA. All RT-PCR plates included two non-template controls (nuclease-free water) and two positive controls using synthetic WNV RNA VR-3274SD purchased from the American Type Culture Collection (Manassas, VA, USA). RT-PCR cycle conditions were 40 min at 50 °C (reverse transcription step), RT enzyme inactivation 2 min at 95 °C, followed by 2-step cycling (40 cycles): denaturation 5 s at 95 °C and combined annealing/extension 30 s at 60 °C. Sample results were defined as: (i) WNV RNA-positive if amplification occurred at or before 35 cycles, (ii) WNV RNA inconclusive if amplification occurred between > 35 and < 40 cycles, and (iii) negative if no amplification occurred at ≥ 40 cycles. All samples were run in triplicate.

## Results

### Raptor results

**Fig. 1:**
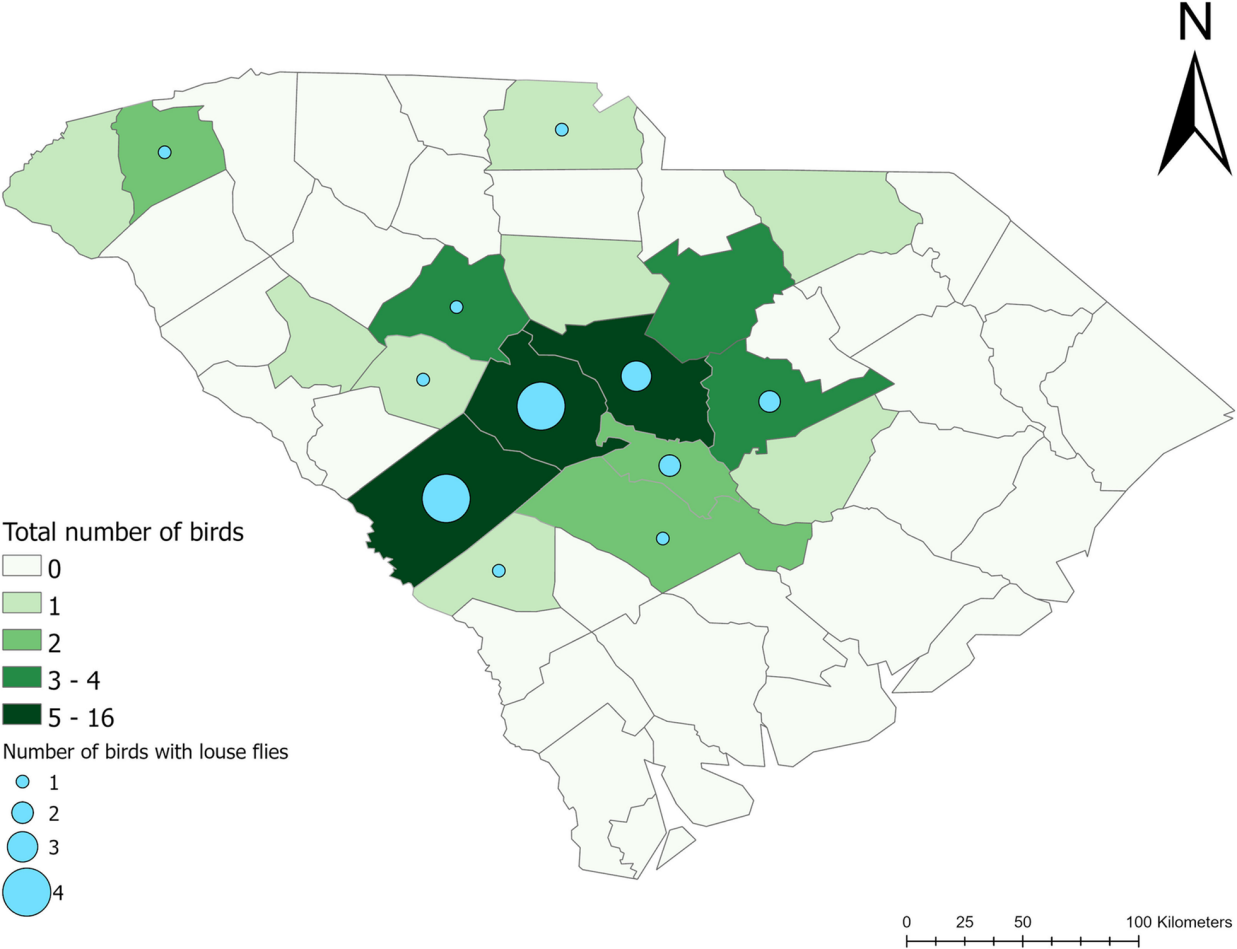
Distribution of origins of raptor and louse fly samples from South Carolina, August 2023 to December 2024

Between August 2023 and December 2024, 71 injured raptors were brought to the Riverbanks Zoo and Garden rehabilitation clinic, representing 14 birds of prey species. Injured raptors were brought to the clinic by private citizens from 18 counties across South Carolina (Fig. 1)—one raptor originated from Stewart County, GA. Richland, Lexington, and Aiken Counties in South Carolina yielded the highest number of injured raptors and louse flies. Of these 71 injured raptors, 29.5% (*n* = 21) had attached louse flies. Raptors with attached louse flies represented six species: Cooper’s hawk (*Astur cooperii*), red-tailed hawk (*Buteo jamaicensis*), red-shouldered hawk (*Buteo lineatus*), broad-winged hawk (*Buteo platypterus*), eastern screech owl (*Megascops asio)*, and a barred owl (*Strix caria*). Of all raptors with attached louse flies, red-tailed hawks represented the most heavily infested avian species.

Intake medical examinations reported a range of clinical phenotypes: grounded with no obvious injuries, infection wounds, emaciation, fractures, documented general trauma, being hit by a car, and neurological abnormalities. Louse flies attached throughout the avian host body, including on the wings, tail, neck, chest, and abdomen, and/or attached throughout the entire body—no apparent preferred corporal attachment location was noted. The number of louse fly attachments ranged from one to seven specimens per raptor, with an average of 2.3 attached specimens per raptor.

### Louse fly and mite results

**Fig. 2:**
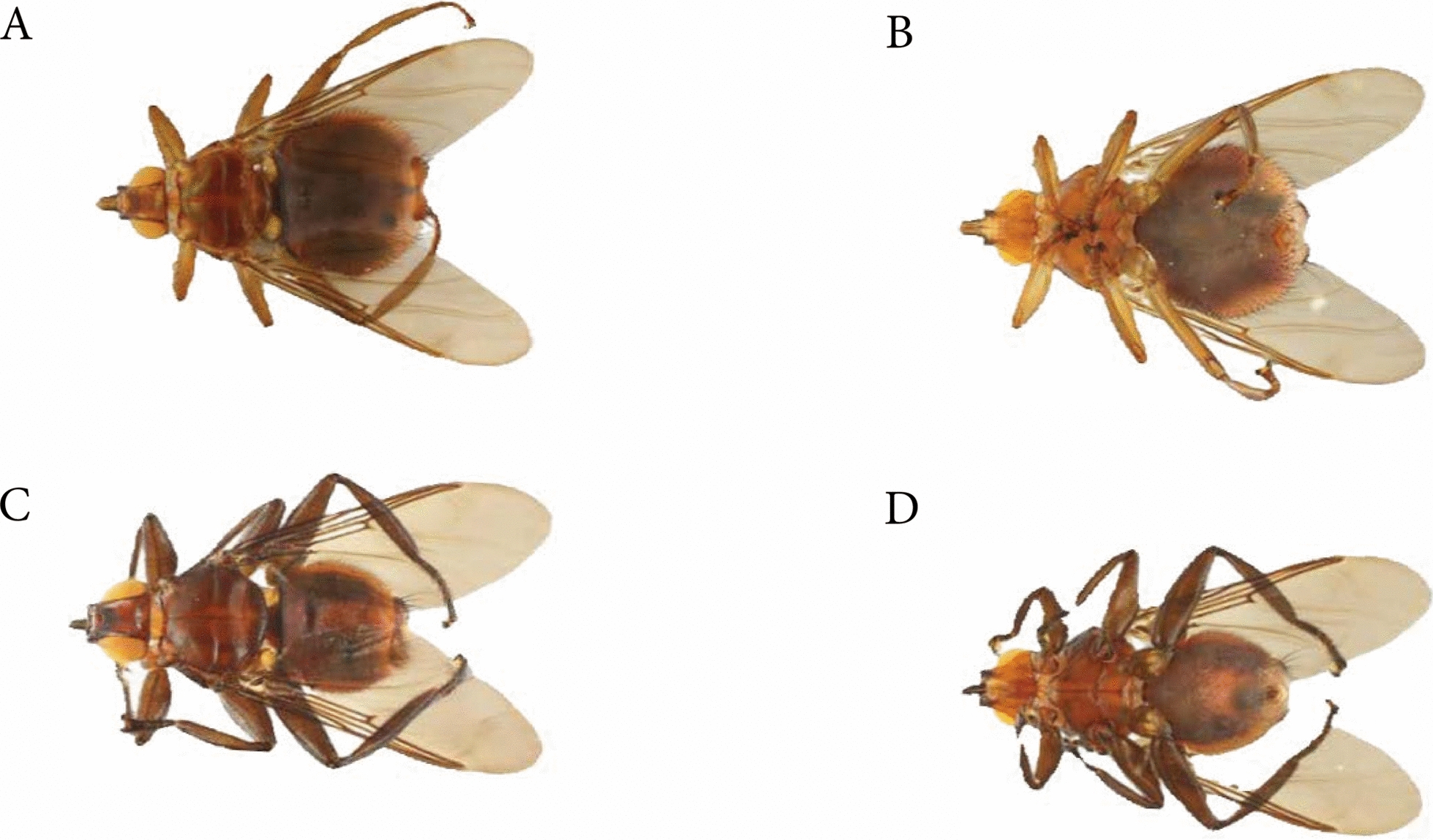
Photomicrographs of louse flies **A** *Icosta americana*, dorsal side; **B** *I. americana*, ventral side; **C** *Icosta rufiventris*, dorsal side **D** *I. rufiventris*, ventral side

During the study period, 75 louse flies were collected for further analysis. Of these, 61 louse flies were identified to species: *Icosta americana* (*n* = 59) and *Icosta rufiventris* (*n* = 2) (Fig. 2)*. Icosta americana* were collected from red-tailed hawks, barred owls, Cooper’s hawks, red-shouldered hawks, and broad-winged hawks. *Icosta rufiventris* were collected from a single eastern screech owl. Of all 36 louse fly pools tested from 2023 to 2024, 42% of these pools tested WNV RNA-positive—all from *I. americana*.

Different mite species were found attached to collected louse flies. Eight of our *I. americana* flies had *Myilages* n.sp. nr. *borealis* (Sarcoptiformes: Epidermoptidae) mites attached. Feather mite clusters were also present on *I. americana*, but were unidentifiable to species.

### Paired WNV RT-PCR results

Saliva and louse flies from 12 different raptors were collected and tested for WNV via RT-PCR. Of these, four raptors (33%) tested WNV-positive, three (25%) of the louse fly pools tested WNV-positive, and three (25%) louse flies tested equivocal (or indeterminate) for WNV (Table 1). Raptor-louse fly pool paired WNV results were mixed; two WNV-positive raptors also had WNV-positive attached louse flies, one WNV-positive raptor had WNV-negative louse fly pools, one WNV-positive raptor had WNV-equivocal louse fly pools, and two WNV-negative raptors had WNV-positive or equivocal louse fly pools (Supplemental Fig. 1). Paired samples were collected from early June to late November (Epi weeks 23—48), and most WNV-positive birds were identified in mid- to late October (Epi weeks 41—44). Lastly, WNV-positive raptors included red-tailed hawks and Cooper’s hawks—no barred owls or red-shouldered hawks were WNV-positive.

**Table 1:** RT-PCR WNV test results for paired raptor and louse fly samples collected from the Riverbanks Zoo and Garden in 2024

Bird number	Species	Collection date (DD/MM/YY)	No. louse fly pools	Bird saliva PCR result	Louse fly pool PCR results		
					PCR (+) % (*n*)	PCR equivocal % (*n*)	PCR (−) % (*n*)
1	Red-tailed hawk	06/07/24	1	(−)	0	0	100% (1)
2	Red-tailed hawk	08/06/24	2	(−)	0	0	100% (2)
3	Barred owl	08/08/24	1	(−)	0	0	100% (1)
4	Cooper’s hawk	08/08/24	2	( +)	100% (2)	0	0
5	Barred owl	10/1/24	1	(−)	0	100% (1)	0
6	Red-tailed hawk	10/10/24	1	( +)	0	0	100% (1)
7	Red-tailed hawk	10/17/24	2	( +)	0	50% (1)	50% (1)
8	Red-tailed hawk	10/28/24	4	( +)	50% (2)	0	50% (2)
9	Barred owl	10/31/24	1	(−)	0	0	100% (1)
10	Red-tailed hawk	11/21/24	3	(−)	100% (3)	0	0
11	Barred owl	11/25/24	2	(−)	0	50% (1)	50% (1)
12	Red-shoulder hawk	11/30/24	1	(−)	0	0	100% (1)

(+) indicates a positive test result [Ct value ≤ 35], “Equivocal” indicates an inconclusive test result [Ct value > 35 and < 40], (−) indicates a negative test result [Ct value ≥ 40], and (*n*) indicates the number of louse fly pools per PCR result

## Discussion

This is the first report of *I. rufiventris* in South Carolina and the broader Southeastern USA to our knowledge. *Icosta rufiventris* is rarely reported in North America compared with other *Icosta* species; however, its identification can be hindered by a lack of resources and images for this species [17]. To promote future studies, one of the *I. rufiventris* specimens was pinned and vouchered at the Smithsonian Museum Support Center (Suitland, MD).

Here, we expand on the known host distributions of *I. americana* and *I. rufiventris* across bird anatomy and their potential role in arboviral maintenance. By the time the birds were examined, *I. americana* did not exhibit a clear preference for host-attachment location. There are no prior published reports of body location preference in most bird-feeding Hippoboscids [9], although the common swift louse fly, *Crataerina pallida*, has been explicitly reported to preferentially feed on the lower rump area of nestling avian hosts and the belly and back of adult avian hosts [18]. Most *I. americana* were not attached and were seen wandering among the feathers, making the feeding sites difficult to determine. The fact that multiple *Icosta* specimens were found distributed across raptor bodies may indicate a lower potential for co-feeding to serve as a transmission mechanism for the WNV-positive specimens. This may strengthen the possibility that *I. americana* can serve as an alternative WNV vector. However, we do acknowledge the injured raptors were handled in various ways prior to arriving at the facility, and it is unclear what effect this has on the distribution and movement of Hippoboscids on the body.

Consistent with prior studies, some of our *Icosta* were hyperparasitized [4, 19]. Epidermoptid mites were found attached to eight louse flies from a single red-tailed hawk. The hyperparasitic relationship between epidermoptid mites and their louse fly hosts is well known in the literature, with mites of the genus *Myialges* Trouessart (1906) found on the abdomen, while mites of the genus *Promyialges* Fain (1965) are located only on the wings [14]. Feather mites have not previously been reported to use louse flies as a major transmission mode among birds [20]. Other mites use Hippoboscid flies for dispersion and oviposition [21]. Future work is needed to elucidate the biological implications of mites exploiting Hippoboscids and any potential influence on raptor host-*Icosta* WNV infection transmission.

*Icosta americana*'s role in WNV transmission and sylvatic cycle maintenance is controversial [5, 6]. In this study, we found discrepancies in WNV RNA detection between raptor hosts and attached louse flies—not all WNV-positive raptors had positive *I. americana*, and vice versa. Riverbanks Zoo and Garden staff reported it is uncommon to find louse flies fully attached or actively feeding on birds when they are brought into rehabilitation clinics. Despite this, louse flies rarely spend time away from their host [22]. If a louse fly leaves its host, it typically returns to its host or to a nearby available host [22]. The movement of louse flies between raptors, particularly from sick or dying birds to healthy ones, could provide a novel transmission mechanism for WNV from infected to uninfected birds if *Icosta* species are competent arboviral vectors. Although outside the scope of the current study, future studies should explore the potential influence of parasite accumulation on weakened animals and birds.

The observed discordance in the presence of WNV RNA in host and parasite suggests that louse flies may be capable vectors. However, we have no information on the virus's status in these flies or where it is found in them. For example, viral RNA remains detectable in fresh blood meals even when the fly lacks vector competence. Additionally, even if *I. americana* could sustain the virus, there are still midgut and salivary barriers to transmission that have not been tested. Yet, some barriers, such as the midgut barrier to disseminated infection, can be overcome by feeding on a host co-infected with filaria [23]. Again, more research on veritable vectorial capacity is warranted to effectively measure the role *I. americana* may play in WNV transmission and ecology.

Some limitations within this study are worth noting. Initial samples stored in ethanol may have led to RNA degradation; however, we did detect WNV viral material in ethanol-stored specimens. Raptor-attached *Icosta* specimens collected in 2023 did not have paired raptor salivary material for vector competency testing, as the decision to collect paired salvia/ectoparasites was made after WNV had been detected in early *I. americana* pools. Thus, the overall number of paired samples was small and should be interpreted with caution. Lastly, no information on WNV status of birds was obtained for samples collected during the August 2023–June 2024 period, limiting our understanding of potential ornithological health impacts. This study still yielded important results and generated proof-of-concept data indicating that Hippoboscid flies should be considered in future WNV ecological or laboratory work.

## Conclusions

From August 2023 to December 2024, 73 *I. americana* and 2 *I. rufiventris* were collected from injured raptors brought into a regional raptor rehabilitation center in South Carolina. Louse flies were attached across the birds’ external anatomy, without any apparent attachment location preference. WNV RNA was detected in 42% of louse fly pools and 33% of tested raptors. WNV infection discordance between raptors and louse fly pools was noted, emphasizing the need for further investigation into louse fly-raptor interactions, and reinforcing the importance of considering louse flies as potential disease vectors within a global One Health context.

## Supplementary Information

Supplementary Material 1.

## Data Availability

The dataset supporting the conclusions of this article is included within the article.
