# Supplementary figures and images for: West Nile virus detected in louse flies (Diptera: Hippoboscidae) collected from rehabilitated raptors in South Carolina
Source: Parasit Vectors. 2026 May 5;19:262. doi: 10.1186/s13071-026-07428-8 (PMC13288572; doi:10.1186/s13071-026-07428-8)

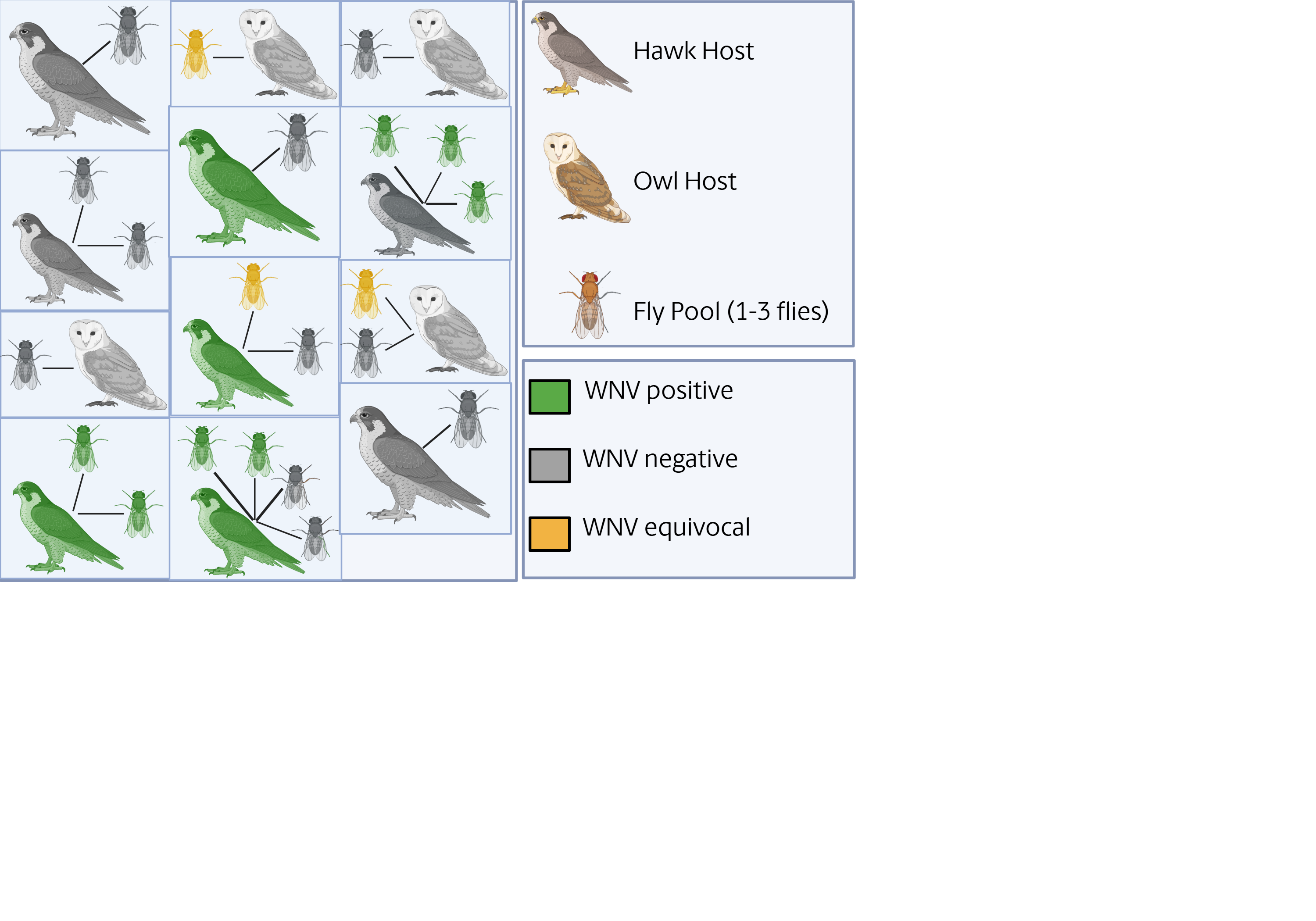

Supplement: Supplementary file 1 — Supplementary Material 1. [file 13071_2026_7428_MOESM1_ESM.png]
